# Sniffing Out the Right Address

**DOI:** 10.1371/journal.pbio.0060134

**Published:** 2008-05-27

**Authors:** Rachel Jones

Olfactory systems in organisms ranging from invertebrates to mammals distinguish between odors using an array of olfactory neurons that express different olfactory receptors—usually just one receptor in a given neuron. Since an organism's genome can encode hundreds of olfactory receptors, researchers have struggled to understand how a single olfactory neuron decides which receptor to express. In Drosophila, it seems that regulatory elements upstream of olfactory receptor genes act like zip codes to direct the expression of each gene to the appropriate class of olfactory neurons.

Anandasankar Ray and colleagues looked at one of the two olfactory organs in fruit flies—the maxillary palp. Unlike the antennae, the maxillary palp contains just six classes of olfactory neurons and seven olfactory receptors (one class of neuron expresses two receptors). This simplicity made it easier for the authors to study, and by comparing the genomes of different Drosophila species, Ray et al. identified gene-specific regulatory elements upstream of these olfactory receptor genes that were highly conserved across species, thereby indicating their likely importance.

By creating fusion genes between these regulatory stretches of DNA and a reporter gene, *GAL4* (which encodes a yeast transcription factor that binds to upstream activating sequences and activates the green fluorescent protein reporter gene), Ray et al. showed that these regulatory regions are necessary for neuron-specific expression; when mutated, they could no longer cause genes to be expressed in olfactory neurons. The authors also showed that most of these regulatory elements in multiple copies could drive expression in the olfactory neurons of the maxillary palps. For example, the olfactory receptor gene *Or46a* is expressed in a type of neuron called pb2B. When the conserved region of DNA upstream of *Or46a* was mutated, the expression of the paired *GAL4* marker was lost.

**Figure:**
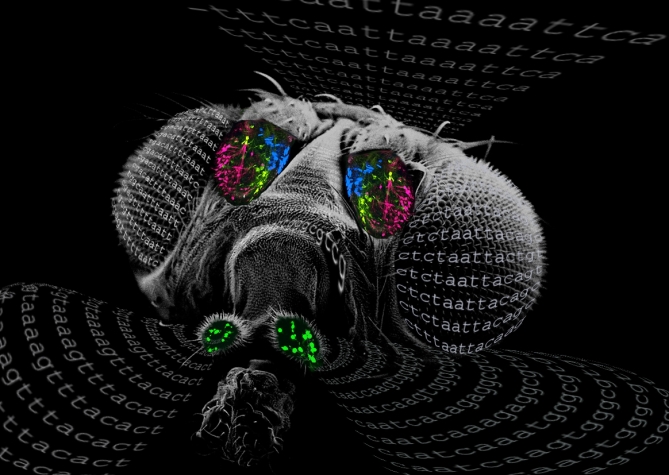
Scanning electron micrograph of a Drosophila head overlaid with expression patterns of four odor receptors in the antenna (blue, yellow, and magenta) and the maxillary palp (green). Sequences represent *cis* elements conserved upstream of odor receptor genes across 12 Drosophila species. (Credits: SEM, Jennifer Perry; Design, Woodstock Tom)

Not all of the regulatory elements carried out this kind of “positive regulation”—some were negative regulators. One olfactory receptor gene, *Or42a*, had two highly conserved upstream elements, and mutation of one of these resulted in the expression of a reporter gene in two types of olfactory neurons, rather than just one. It seems that upstream regulatory sequences of this receptor drive expression in both types of neurons, but the negative regulator suppresses expression in neurons other than the appropriate class. Another gene, *Or59c*, is close to a binding site for a known transcription factor (Scalloped or Sd) that is expressed in olfactory organs. Functional analysis showed that Scalloped seems to repress expression of *Or59c* in the neighboring neurons

So it appears that the neuron-specific expression of olfactory receptor genes in the maxillary palp depends on an interplay between positive and negative regulatory elements that are specific to each gene. The authors liken these elements to molecular zip codes that specify the neuronal “address” at which each gene should be expressed. The cross-species comparison showed that these regulatory elements, and the organization of gene expression as well as the odor responses of neurons in the maxillary palp, have been conserved for tens of millions of years since the species in question diverged—even though the amino acid sequences of the olfactory receptors themselves have not been highly conserved. This indicates that the maxillary palp may be important for odor discrimination and olfactory behavior in Drosophila.

Interestingly, some of these regulatory elements are also found upstream of genes that are involved in axon guidance, suggesting that they might contribute to the formation of connections between olfactory neurons and neurons in the antennal lobes that receive these connections. So as well as directing the expression of specific olfactory receptors to specific olfactory neurons, these regulatory elements might also control the formation of an olfactory “map” higher in the nervous system.

